# Genomic Analysis of Ciprofloxacin-Resistant *Salmonella enterica* Serovar Kentucky ST198 From Spanish Hospitals

**DOI:** 10.3389/fmicb.2021.720449

**Published:** 2021-10-05

**Authors:** Xenia Vázquez, Javier Fernández, Margarita Bances, Pilar Lumbreras, Miriam Alkorta, Silvia Hernáez, Elizabeth Prieto, Pedro de la Iglesia, María de Toro, M. Rosario Rodicio, Rosaura Rodicio

**Affiliations:** ^1^Área de Microbiología, Departamento de Biología Funcional, Universidad de Oviedo (UO), Oviedo, Spain; ^2^Instituto de Investigación Sanitaria del Principado de Asturias (ISPA), Oviedo, Spain; ^3^Servicio de Microbiología, Hospital Universitario Central de Asturias (HUCA), Oviedo, Spain; ^4^Research and Innovation, Artificial Intelligence and Statistical Department, Pragmatech AI Solutions, Oviedo, Spain; ^5^Laboratorio de Salud Pública (LSP) del Principado de Asturias, Dirección General de Salud Pública, Oviedo, Spain; ^6^Servicio de Microbiología, Hospital Universitario Donostia (HUD)-IIS Biodonostia, San Sebastián, Spain; ^7^Servicio de Microbiología, Hospital Universitario de Álava (HUA), Vitoria-Gasteiz, Spain; ^8^Servicio de Microbiología, Hospital Universitario San Agustín, Avilés, Spain; ^9^Servicio de Microbiología, Hospital Universitario de Cabueñes, Gijón, Spain; ^10^Plataforma de Genómica y Bioinformática, Centro de Investigación Biomédica de La Rioja (CIBIR), Logroño, Spain; ^11^Departamento de Bioquímica y Biología Molecular, Universidad de Oviedo (UO), Oviedo, Spain

**Keywords:** *Salmonella enterica* serovar Kentucky, ST198, SGI1-K, IS*26*, fluoroquinolone resistance, multidrug resistance, whole genome sequencing, phylogenetic analysis

## Abstract

*Salmonella enterica* serovar Kentucky (*S*. Kentucky) with sequence type (ST) 198 and highly resistant to ciprofloxacin (ST198-Cip^*R*^) has emerged as a global MDR clone, posing a threat to public health. In the present study, whole genome sequencing (WGS) was applied to characterize all Cip^*R*^ *S.* Kentucky detected in five Spanish hospitals during 2009–2018. All Cip^*R*^ isolates (*n* = 13) were ST198 and carried point mutations in the quinolone resistance-determining regions (QRDRs) of both *gyrA* (resulting in Ser83Phe and Asp87Gly, Asp87Asn, or Asp87Tyr substitutions in GyrA) and *parC* (with Thr57Ser and Ser80Ile substitutions in ParC). Resistances to other antibiotics (ampicillin, chloramphenicol, gentamicin, streptomycin, sulfonamides, and tetracycline), mediated by the *bla*_*TEM–*__1__*B*_, *catA1, aacA5*, *aadA7, strA*, *strB*, *sul1*, and *tet*(A) genes, and arranged in different combinations, were also observed. Analysis of the genetic environment of the latter resistance genes revealed the presence of multiple variants of SGI1 (*Salmonella* genomic island 1)-K and SGI1-P, where all these resistance genes except *catA1* were placed. IS*26* elements, found at multiple locations within the SGI1 variants, have probably played a crucial role in their generation. Despite the wide diversity of SGI1-K- and SGI1-P-like structures, phylogenetic analysis revealed a close relationship between isolates from different hospitals, which were separated by a minimum of two and a maximum of 160 single nucleotide polymorphisms. Considering that *S. enterica* isolates resistant to fluoroquinolones belong to the high priority list of antibiotic-resistant bacteria compiled by the World Health Organization, continuous surveillance of the *S*. Kentucky ST198-CIP^*R*^ clone is required.

## Introduction

*Salmonella enterica* is one of the major causes of bacterial gastrointestinal infections in humans, worldwide, with estimates of 93.8 million cases each year and 155,000 deaths (Majowicz et al., 2010). In the European Union (EU), after a long period of declining trend, the number of cases of human salmonellosis has stabilized over the past 5 years (European Food Safety Authority [EFSA] and European Centre for Disease Prevention and Control [ECDC], 2021). Along this period, salmonellosis remained the second most frequent food-borne zoonosis (only preceded by campylobacteriosis), with a total of 87,923 confirmed cases and a notification rate of 20.0 cases per 100,000 inhabitants reported in 2019 (European Food Safety Authority [EFSA] and European Centre for Disease Prevention and Control [ECDC], 2021). In Spain, from 2015 to 2018, the number of confirmed cases of human salmonellosis ranged between 8,730 (2018) and 9,818 (2016), while neither the complete data for 2019 nor the rate per 100,000 inhabitants are available (European Food Safety Authority [EFSA] and European Centre for Disease Prevention and Control [ECDC], 2021).

Human salmonellosis is usually a self-limiting infection that remains confined to the intestine and resolves in about one week, even in the absence of antimicrobial treatment. Nevertheless, invasive, severe infections may occur in immunocompromised patients, as well as in small children and the elderly, in which case the treatment may become life-saving. The high rate of resistance against traditional antimicrobials has prompted the use of newer broad-spectrum drugs, like third generation cephalosporins and fluoroquinolones (Hohmann, 2001), which are recommended by therapeutic international and national (including Spanish) guidelines as first choices for *Salmonella* severe infections (Gilbert et al., 2021; Mensa and Soriano, 2021). These compounds are listed by the World Health Organization (WHO) as “critically important antimicrobials” with the highest priority for human medicine (World Health Organization [WHO], 2016). Fortunately, resistance to cephalosporins remains low in *Salmonella* isolates recovered from humans (1.5 and 1.2% for cefotaxime and ceftazidime, respectively), food-animals and foods in the EU (European Food Safety Authority [EFSA] and European Centre for Disease Prevention and Control [ECDC], 2020). However, the proportion of human isolates resistant to ciprofloxacin, a second generation (2a) fluoroquinolone (Pham et al., 2019), was of 12.5% on average, with extremely high levels reported for certain serovars, particularly in *S. enterica* serovar (*S*. Kentucky; 85.7%) (European Food Safety Authority [EFSA] and European Centre for Disease Prevention and Control [ECDC], 2020). In *S*. Kentucky, resistance to ampicillin, sulfonamides, tetracyclines (also common in other *S. enterica* serovars) and gentamicin (infrequently found in *Salmonella*) are very high as well, leading to multidrug resistance (MDR).

The high frequency of MDR in *S*. Kentucky has been associated with the expansion of a single clone with sequence type (ST) 198 and belonging to *Xba*I-pulsed field gel electrophoresis cluster X1 (Weill et al., 2006; Le Hello et al., 2011, 2013a; Hawkey et al., 2019). Phylogenomic analysis indicated that this clone emerged in Egypt around 1989, linked to the acquisition of a variant of *Salmonella* genomic island 1 (SGI1-K), conferring resistance to multiple antibiotics, such as ampicillin, streptomycin, gentamicin, sulfonamides, and tetracycline (Le Hello et al., 2013a; Hawkey et al., 2019). The SGI1-K prototype (48.7 Kb) consists of a 27 kb backbone and a complex resistance region encompassing a In*4*-type integron, parts of the Tn*21*, Tn*1721*, Tn*5393*, and Tn*2* transposons, and two copies of IS*26* flanking the defective Tn*2* in opposite orientation (Hamidian et al., 2015). SGI1-K is inserted within the *S*. Kentucky chromosome, between the *trmE* (*thdF*) and *yidY* genes, with the resistance region flanked by *resG* and Δ*S044* pertaining to the island backbone (Hamidian et al., 2015). Multiple variants of SGI1-K have previously been reported, including the highly degenerated SGI1-P and SGI1-Q structures, which confer only resistance to ampicillin or lack antibiotic resistance genes, respectively (Doublet et al., 2008; Le Hello et al., 2011; Hawkey et al., 2019).

After acquisition of SGI1-K by *S*. Kentucky, the already MDR clone accumulated various mutations in the quinolone-resistance-determining regions (QRDRs) of genes encoding subunits of the target DNA gyrase (*gyrA*) and DNA topoisomerase IV (*parC*) (Le Hello et al., 2013b), which combined are responsible for resistance to ciprofloxacin. The Ser83Phe substitution in GyrA, which by itself confers resistance to nalidixic acid (Hamidian et al., 2015), was the first to occur, followed in time by the Ser80Ile substitution in ParC which, together with the former, increased the minimum inhibitory concentration (MIC) of ciprofloxacin. Nonetheless, high-level resistance only emerged after the advent of additional mutations in *gyrA*-87, and this was accompanied by clonal expansion of *S*. Kentucky ST198-Cip^*R*^, and its subsequent spread from Egypt to many other geographical regions, including Europe (Le Hello et al., 2011; Hawkey et al., 2019). A second mutation found in the *parC* gene (Thr57Ser) has also been reported in *S*. Kentucky ST198 detected in French travelers returning from Africa, in a human patient in United States, as well as in retail chicken carcasses from Egypt (Weill et al., 2006; Ramadan et al., 2018; Shah et al., 2018). Such change, however, does not appear to be associated with quinolone resistance, as it was also identified in isolates susceptible to nalidixic acid (Weill et al., 2006). Although alterations in the target topoisomerases are the main cause of ciprofloxacin resistance in *S.* Kentucky, the involvement of the major multidrug AcrAB-TolC efflux pump, and of mutations affecting the *rpoB* gene coding for the β-subunit of the enzyme RNA polymerase, have also been reported (Weill et al., 2006; Baucheron et al., 2013; Brandis et al., 2021). Many other efflux pumps were identified in *S. enterica* (Li et al., 2018), but their contribution to high level CIP^*R*^ in *S*. Kentucky has yet to be demonstrated.

Taking into account (i) that *S. enterica* resistant to fluoroquinolones is amongst the high priority pathogens listed by WHO (Tacconelli et al., 2018), (ii) that *S*. Kentucky ST198-Cip^*R*^ represents an emerging threat to food safety and public health, and (iii) that ciprofloxacin is a treatment of choice for severe infections caused by *S. enterica* in adults, the present study applied whole genome sequence analyses to thoroughly characterize and compare ciprofloxacin resistant isolates of this serovar, which were recovered in recent years at different hospitals located in Northern Spain. Particular attention was paid to the genetic environment of their antimicrobial resistance genes, and the mobile genetic elements associated with them.

## Materials and Methods

### Bacterial Isolates and Antimicrobial Susceptibility Testing

A total of 13 isolates of *S*. Kentucky resistant to ciprofloxacin were analyzed. They were recovered between 2009 and 2018 from fecal samples of patients with gastroenteritis, attended at five Spanish hospitals or primary care centers associated with them (Table 1). Stool samples were cultured using selective media, such as selenite broth and Hecktoen agar (bioMerieux, Marcy l’Etoile, France), and subsequently identified by MALDI-TOF (Bruker Daltonics, Billerica, MA). Experimental serotyping of the isolates was performed either at the Spanish National Center of Microbiology (Madrid) or directly at the hospital. Susceptibility to antimicrobial agents was determined by automated MicroScan NC 53 (Beckman Coulter, Brea, CA, United States), and complemented with disk diffusion assays using Mueller-Hinton agar and commercially available discs (Oxoid, Madrid, Spain). Results were interpreted according to the Clinical and Laboratory Standards Institute Guidelines (Clinical and Laboratory Standards Institute [CLSI], 2019). MICs to ciprofloxacin were determined by Etest (bioMérieux, Marcy l'Étoile, France).

**TABLE 1:** Origin and resistance properties of *Salmonella enterica* serovar Kentucky ST198 isolates from Spanish hospitals.

**Isolate^a^**	**Travel history^b^**	**Resistance phenotype^c^**	**SGI1-K (SGI1-P) genes** Other genes	**CIP MIC (μg/mL)**	**Amino acid substitutions**		**Plasmid Inc (size in bp)^d^**
					**GyrA**	**ParC**	
LSP 213/09	na	CHL, TET, NAL, CIP	***tet*(A),** *catA1*, *aac(6′)-Iaa*	8	Ser83Phe Asp87Gly	Thr57Ser Ser80Ile	ColpVC (4,110)
LSP 150/10	Morocco	AMP, GEN, STR, SUL, TET, NAL, CIP	***bla*_*TEM–1B*_, *aacA5*, *aadA7*, *sul1*, *tet*(A)**, aac(6′)-Iaa	12	Ser83Phe Asp87Asn	Thr57Ser Ser80Ile	ColE (5,058); Col156 (5,769); nid (10,524)
LSP 105/15	na	AMP, NAL, CIP	***bla*_*TEM–1B*_**, *aac(6′)-Iaa*	16	Ser83Phe Asp87Asn	Thr57Ser Ser80Ile	nd
LSP 235/17	na	TET, NAL, CIP	***tet*(A),** *aac(6′)-Iaa*	>32	Ser83Phe Asp87Asn	Thr57Ser Ser80Ile	nid (3,893; 4,631)
LSP 314/17	Bali	AMP, GEN, STR, SUL, TET, NAL, CIP	***bla*_*TEM–1B*_, *aacA5*, aadA7, *strA*, *strB*, *sul1*, *tet*(A),** *aac(6′)-Iaa*	12	Ser83Phe Asp87Asn	Thr57Ser Ser80Ile	nd
HUD 1/09	Tanzania	GEN, STR, SUL, TET, NAL, CIP	***aacA5***, ***aadA7***, ***strB***, ***sul1***, ***tet*(A),** *aac(6)-Iaa*	6	Ser83Phe Asp87Tyr	Thr57Ser Ser80Ile	nd
HUD 2/09	South Africa	AMP, GEN, STR, SUL, TET, NAL, CIP	***bla*_*TEM–1B*_, *aacA5*, *aadA7*, *strB***, ***sul1*, tet(A),** aac(6)-Iaa	8	Ser83Phe Asp87Tyr	Thr57Ser Ser80Ile	nd
HUD 1/13	nth	AMP, NAL, CIP	***bla*_*TEM–1B*_,** *aac(6)-Iaa*	8	Ser83Phe Asp87Asn	Thr57Ser Ser80Ile	nid (1,145)
HUD 1/14	nth	AMP, GEN, STR, SUL, TET, NAL, CIP	***bla*_*TEM–1B*_, *aacA5, aadA7*, *sul1*, *tet*(A),** *aac(6)-Iaa*	12	Ser83Phe Asp87Asn	Thr57Ser Ser80Ile	ColE (4,132); nid (3,372; 4,010)
HUD 1/15	nth	AMP, GEN, STR, SUL, TET, NAL, CIP	***bla*_*TEM–1B*_, *aacA5, aadA7, sul1, tet*(A),** *aac(6)-Iaa*	12	Ser83Phe Asp87Asn	Thr57Ser Ser80Ile	ColE (2,504); nid (3,371; 3,904; 4,179)
HUD 1/17	Morocco	TET, NAL, CIP	***tet*(A),** *aac(6)-Iaa*	8	Ser83Phe Asp87Asn	Thr57Ser Ser80Ile	ColE (2,448); nid (4,110)
HUA 3/18	Morocco	AMP, GEN, STR, SUL, TET, NAL, CIP	***bla*_*TEM–1B*_, *aacA5*, *aadA7*, *sul1*, *tet(*A),** *aac(6)-Iaa*	12	Ser83Phe Asp87Asn	Thr57Ser Ser80Ile	ColE (4,020); nid (2,117; 3,985)
HUA 10/18	nth	SUL, TET, NAL, CIP	***sul1*, *tet*(A)**, *aac(6)-Iaa*	8	Ser83Phe Asp87Asn	Thr57Ser Ser80Ile	IncI1 (85,307); ColE (4,105); nid (2,185; 3,985; 4,164; 5,413)

*^a^Isolates are designated with the initials of the center which supplied them, followed by a serial number/last two numbers of the year of recovery. LSP, Laboratory of Public Health of the Principality of Asturias, acting as regional reference center for *Salmonella*. LSP isolates come from “Hospital Universitario Central de Asturias,” Oviedo, Asturias (LSP 213/09 and LSP 105/15), “Hospital Universitario de Cabueñes,” Gijón, Asturias (LSP 150/10 and LSP 314/17); and “Hospital Universitario San Agustín,” Avilés, Asturias (LSP 235/17). HUD, “Hospital Universitario Donostia,” Basque Country; HUA, “Hospital Universitario de Álava,” Basque Country.*

*^b^nth, no travel history; na, information not available.*

*^c^AMP, ampicillin; CHL, chloramphenicol; GEN, gentamicin; STR, streptomycin; SUL, sulfonamides; TET, tetracycline, NAL, nalidixic acid; CIP, ciprofloxacin.*

*^d^Inc, incompatibility group; nid, Inc not identified; nd, plasmid(s) not detected.*

### Whole Genome Sequencing and Bioinformatics Analysis

WGS of the isolates was determined by Illumina either at the sequencing facility of the ‘‘Centro de Investigación Biomédica,’’ La Rioja (CIBIR), Spain or Eurofins Genomics (Ebersberg, Germany). Total DNA was extracted from overnight cultures grown in Luria-Bertani (LB) broth, using the GenElute^TM^ Bacterial Genomic DNA Kit (Sigma-Aldrich; Merck Life Science, Madrid, Spain), according to the manufacturer’s instructions. Paired-end reads of 100 or 150 nt were sequenced with a HiSeq 2500 or a NovaSeq 6000 S2 PE150 XP, in the case of CIBIR and Eurofins, respectively. Reads were assembled with the VelvetOptimiser.pl script implemented in the ‘‘on line’’ version of PLACNET,^1^ which also served for plasmid reconstruction (Vielva et al., 2017). The quality of the assemblies was evaluated with QUAST (Quality Assessment Tool for Genome Assemblies; Gurevich et al., 2013), and the output information is compiled in Supplementary Table 1. The genomes were deposited in GenBank under accession numbers provided in the same table and also below, and annotated by the NCBI Prokaryotic Genome Annotation Pipeline (PGAP^2^). Several tools from the Center for Genomic Epidemiology (CGE) of the Technical University of Denmark (DTU), such as MLST, ResFinder, PlasmidFinder, and pMLST, were used for bioinformatic analysis.^3^ ResFinder detects chromosomal mutations that mediate antimicrobial resistance (such as mutations in *gyrA*, *gyrB*, *parC*, and *parE* genes involved in quinolone resistance), and identifies acquired genes [including plasmid-mediated quinolone resistance (PMQR) genes, such as *qnr*, *qepA*, *oqxAB*, and *aac(6′)-Ib-cr*], in bacterial genome sequences. After annotation of the genomes, analysis of relevant regions, including SGI1-K-related DNA, DNA encoding efflux pumps and their regulatory proteins (*acrAB*, *acrA*, *acrD*, *acrEF*, *acrR*, *acrS*, *ramR*, *ramA*, *soxR*, *sosS*, *marC*, *marRAB*, and *rob*) and of the *rpoB* gene, was performed with the aid of BLASTn, CLONE Manager (CloneSuit9), and MyDbFinder (CGE, DTU). For the latter, a database comprising all open reading frames from SGI1-K and flanking *orfs* (based on accession number AY463797), was specifically built for this study. The presence and orientation of more than one copy of IS*26* was used as an initial reference for the assembly of contigs belonging to the island in each genome. PCR amplification with the primers compiled in Supplementary Table 2, followed by Sanger sequencing of the obtained amplicons (carry out at STAB VIDA, Caparica, Portugal), were performed when required to reconstruct the intact islands.

### Phylogenetic Analysis

The relationship between the *S*. Kentucky isolates from Spanish hospitals was inferred using the CSI phylogeny tool (version 1.4), available at the CGE website (Kaas et al., 2014). The pipeline was run with default parameters, using the genome of *S*. Kentucky strain 201001922 (accession number CP028357) as reference for SNP calling. Additional genomes of *S*. Kentucky ST198 (see Supplementary Table 3 for accession numbers), were also included in the analysis, and the resulting SNP matrix is shown in Supplementary Table 4. Bootstrap support for the consensus tree relied on 1,000 replicates (Felsenstein, 1985).

### Ethics Approval Statement

This study was approved by the Research Ethics Committee of the Principality of Asturias (Code CEImPA 2020.446).

## Results

### General Properties of the Isolates

All isolates in this study (*n* = 13) derived from human clinical samples analyzed at five hospitals in Northern Spain (Table 1). They were identified as *S.* Kentucky and selected on the basis of MIC values to ciprofloxacin >2 μg/ml. The assembly size of the sequenced genomes ranged from 4.785 Mb (HUD 1/14) to 4.857 Mb (HUA 10/18), and MLST performed *in silico* assigned all isolates to ST198. Plasmids were found in nine of them, in numbers ranging from one (LSP 213/09 and HUD 1/13) up to six (HUA 10/18). A plasmid of 85.3 kb, carried by the latter isolate, belonged to incompatibility group IncI. All other plasmids were smaller than 10.5 kb, and belonged to ColpVC, ColE, Col156, or had an unidentified replicon.

With regard to antimicrobial susceptibility, six distinct resistance phenotypes were identified (Table 1). According to the bases of selection, resistance to ciprofloxacin, and also to nalidixic acid, was common to all isolates. MICs to ciprofloxacin ranged between 6 and >32 μg/ml. Resistances to ampicillin, gentamicin, streptomycin, sulfonamides, and tetracycline, arranged in different combinations, were also observed. Such resistances are expected to be conferred by SGI1-K or variants herein, including SGI1-P, which are characteristically associated with the ST198-Cip^*R*^ clone. The most common phenotype, shared by six isolates, comprised all SGI1-K-encoded resistances. Apart from that, a single isolate (LSP 213/09) was resistant to chloramphenicol, whereas resistances to broad spectrum cephalosporins, carbapenems, or colistin were not detected.

### Genetic Bases of Ciprofloxacin Resistance

The 13 isolates characterized in the present study had four mutations in the QRDR, two in *gyrA* and two in *parC*. One of the *gyrA* mutations, leading to the Ser83Phe substitution in the protein, and the two mutations in *parC*, resulting in Ser80Ile and Thr57Ser replacements, were shared by all isolates. In contrast, three different changes affecting the 87 codon of *gyrA*: Asp87Asn, Asp87Tyr, and Asp87Gly were detected in ten, two, and one isolates, respectively. Travel to an African country (Morocco, Tanzania, and South Africa) or to Bali (Indonesia), prior the onset of the disease, was documented for six patients. For the remaining patients, this was not the case or the information was not available (Table 1). As indicated before, MIC values to ciprofloxacin of the *S*. Kentucky isolates analyzed in the present study ranged from 6 up to >32 μg/ml. Searching additional resistance mechanisms, which could justify the observed differences, revealed single mutations in the *acrR* (encoding a local regulator of the AcrAB-TolC efflux pump) and *marC* (unknown function) genes of LSP 235/17, the isolate with the higher MIC to ciprofloxacin (>32 μg/ml). A change of G→A in each of these genes led to the conservative substitution of Val by Ile in the proteins, so the high MIC value of the isolate is unlikely to be due to the detected mutations. Alterations in structural or regulatory genes (*acrA*, *acrB*, *acrS*, *acrE*, *acrF*, *tolC*, *ramR*, *ramA*, *soxR*, *soxS*, *marR*, *marA*, and *rob*), for other pumps, including their putative promoter regions, or mutations in the *rpoB* gene, were not observed. PMQR genes were neither detected. Thus, the bases for MIC variation in the analyzed isolates remain unknown.

### High Diversity of *Salmonella* Genomic Island 1-K (and *Salmonella* Genomic Island 1-P) in the Clinical Isolates of *Salmonella enterica* Serovar Kentucky

**FIGURE 1:**
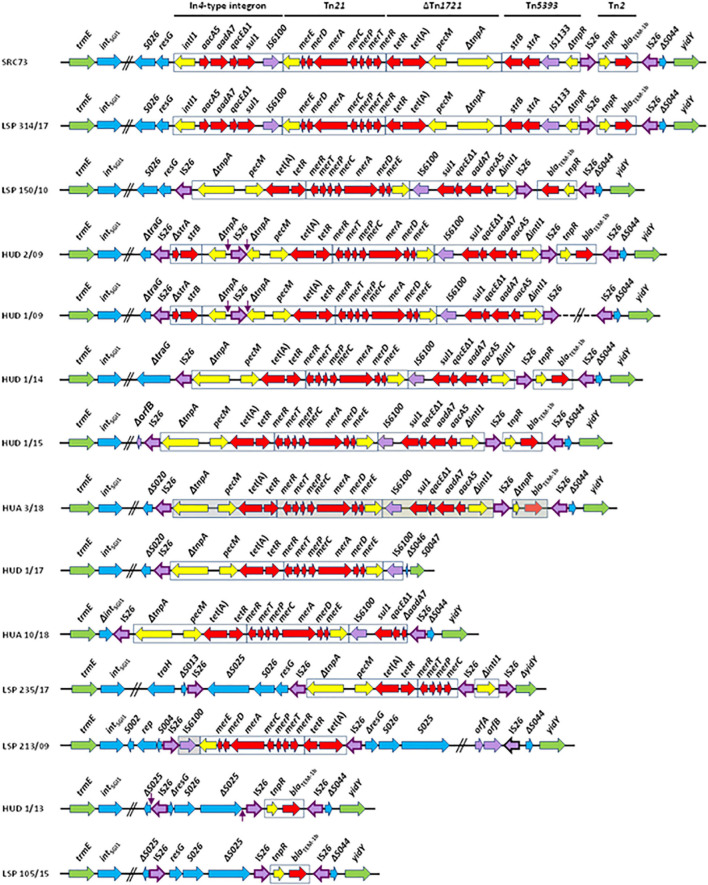
SGI1-K (SGI1-P) variation in *Salmonella enterica* serovar Kentucky ST198-Cip^*R*^ from Spanish hospitals. Coding regions are represented by arrows pointing in the direction of transcription and having different colors according to their function. Green, flanking chromosomal DNA; blue, SGI1 backbone; red, resistance genes; yellow, transposon and integron genes other than resistance genes; purple, insertion sequences with IS*26* highlighted by a darker border. Target site duplications are indicated by vertical arrows. Contiguous horizontal lines crossed by two parallel oblique lines indicate that not all genes are shown. In HUD 1/09, the dashed line crossed by two parallel oblique lines denotes that the corresponding sequence is unknown. LSP, Laboratory of Public Health of the Principality of Asturias, acting as regional reference center for *Salmonella*. LSP isolates come from “Hospital Universitario Central de Asturias,” Oviedo, Asturias (LSP 213/09 and LSP 105/15), “Hospital Universitario de Cabueñes,” Gijón, Asturias (LSP 150/10 and LSP 314/17) and “Hospital Universitario San Agustín,” Avilés, Asturias (LSP 235/17). HUD, “Hospital Universitario Donostia,” Basque Country; HUA, “Hospital Universitario de Álava,” Basque Country.

The diversity of resistance phenotypes shown by the clinical *S*. Kentucky isolates in the present study was mostly associated with a high variability of SGI1-K. As shown in Figure 1, each isolate has a distinct SGI1-K variant, which differed in structure and/or resistance gene content. The genomic island of LSP 314/17 was the only one that coincided with the SGI1-K prototype. Variations observed in all other isolates comprised deletions and inversions of variable size, affecting different components of the resistance region, the SGI1 backbone, and/or the flanking chromosomal DNA at the *yidY* end (in the case of HUD 1/17 and LSP 235/17). The islands of HUD 1/13 and LSP 105/15 were closely related. Both carried a Tn*2*-like transposon with the *bla*_*TEM–*__1_ gene as the only resistance element, and could then be assigned to SGI1-P. When compared with the control SGI1-K, most islands carry a copy of IS*26* between a deleted, and frequently inverted, resistance region, and the upstream SGI1-K backbone, which was also deleted in all but one isolate (LSP 150/10). In three isolates, a copy of IS*26* was found inside the island backbone (LSP 235/17, HUD 1/13, and LSP 105/15), and in one isolate (LSP 213/09) the 5′-end of the backbone was interrupted by the resistance region and the second segment, now contiguous to the 3′-end of the backbone, was inverted. In all, a total of 38 copies of IS*26* were identified, supporting the essential role of this element in the generation of the variants.

Apart from the resistance genes carried by SGI1-K- and SGI-P-like islands, the *catA1* gene was detected in a single isolate (LSP 213/09), resistant to chloramphenicol. The gene was located on a 5,047 bp contig which has to be of chromosomal origin, since the isolate carried a single ColpVC plasmid of 4,110 bp. The contig is flanked by IS*1* and IS*26*, and a 4,917 bp segment, consisting of IS*1*, *catA1*, *tnpA*_*Tn*_*_21_*, and Δ*tnpR*_*Tn*_*_21_*, but excluding IS*26*, is 100% identical to regions found in plasmids, as well as in the chromosome of strains from different serovars of *S. enterica*, including *S*. Typhimurium, *S*. Wien, *S*. Wirchow, *S*. Typhi, and *S*. Paratyphi B (not shown). It is finally of note that all *S*. Kentucky isolates from the present study carried a cryptic *aac(6′)-1aa* gene of chromosomal location, which is widespread in *S. enterica* but fails to confer the resistance phenotype expected for AAC(6′)-Iaa, which effectively acetylates kanamycin, tobramycin, and amikacin (Salipante and Hall, 2003).

### Genomic Relationships Between the Isolates

**FIGURE 2:**
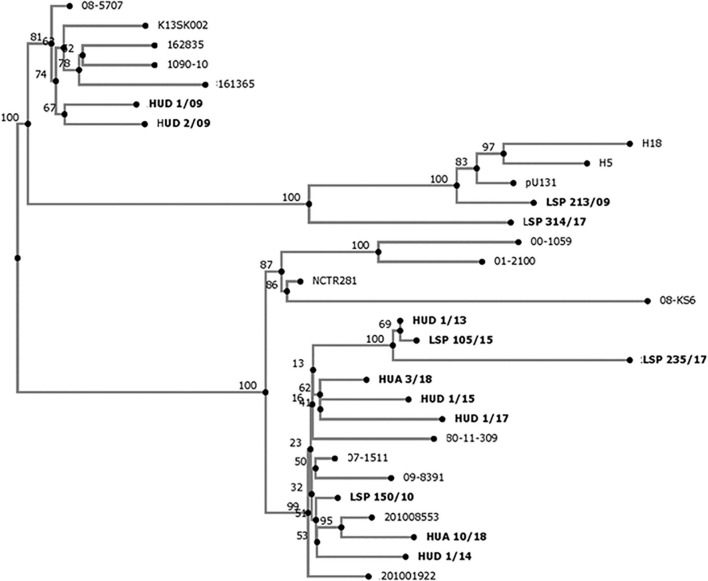
Phylogenetic tree showing the relationships between *Salmonella enterica* serovar Kentucky ST198-Cip^*R*^ isolates from Spanish hospitals (highlighted in bold), and other *S.* Kentucky ST198 isolates. The whole genome single nucleotide polymorphism based analysis was established with the CSI Phylogeny 1.4 (https://cge.cbs.dtu.dk/services/CSIPhylogeny/), using the genome of *S*. Kentucky strain 201001922 (accession number CP028357) as reference. Numbers at the nodes represent bootstrap values based on 1,000 replicates.

As shown in Figure 2, the 13 isolates from Spanish hospitals were closely related, differing by a minimum of two single nucleotide polymorphisms (SNP) (HUD 1/13 versus LSP 105/15), and a maximum of 160 SNP (LSP 213/09 versus LSP 235/17; Supplementary Table 4). However, they could be separated into two clades, one including nine isolates from five hospitals, and the other comprising the remaining four, detected in three hospitals.

## Discussion

In the present study, *S*. Kentucky ST198 resistant to ciprofloxacin and carrying SGI1-K- and SGI1-P-like structures were detected in five hospitals from Northern Spain. Travel to an African country before the onset of the disease was documented for five patients, while in another one the disease could have been acquired during a previous trip to Indonesia. The link between *S*. Kentucky ST198-Cip^*R*^ and Africa is well established, as there is evidence indicating that the clone has emerged in Egypt, from where it has disseminated first into Northern, Southern, and Western Africa, and then into Asia and the European Union (Le Hello et al., 2011, 2012; Hawkey et al., 2019). With regard to Indonesia, two epidemiologically unrelated *S*. Kentucky-Cip^*R*^ isolates were detected in French patients that reported travel to this region in the early 90’ (Le Hello et al., 2012). Interestingly, these isolates did not belong to the *Xba*I pulsotype X1 characteristically associated with African isolates but to pulsotype X2 and they carried the SGI1-J4 and SGI1-J6 islands instead of the SGI1-K-like structures identified in the African epidemic clone (Le Hello et al., 2012). However, *S*. Kentucky ST198-Cip^*R*^ isolates with pulsotype group X1 and SGI1-K have also been found in Indonesia (Le Hello et al., 2013a, b). In the present study, the Spanish isolate from a patient with previous travel history to Bali (LSP 314/17) was the only one containing the canonical SGI1-K, and probably belongs to the African clone. Interestingly, four patients did not report travel to a foreign country prior to the onset of the disease, consistent with intra-national spread of the ST198-Cip^*R*^ clone.

SGI1-K was first reported in *S*. Kentucky SRC73, isolated in 2001 from spice imported into Australia from India and, since then, multiple variants have been detected (Levings et al., 2007; Doublet et al., 2008; Le Hello et al., 2011; Hawkey et al., 2019). Most if not all of those described herein could have been generated by the activity of IS*26*, an insertion sequence which is playing a key role in the evolution of complex resistance regions. IS*26* is well suited for this by using two mechanisms of movement: (i) the copy-in mechanism, which requires DNA replication and results in duplication of both IS*26* and 8 bp originally present at a randomly selected target site; and (ii) the targeted conservative mechanism, which involves two copies of IS*26* and occurs in the absence of IS*26* replication or target site duplication (TSD) (Harmer et al., 2014, 2020; He et al., 2015). In the SGI1-K-like structures analyzed in this study, the number of IS*26* elements ranged from one (HUD 1/17) up to four (HUD 2/09, HUD 1/09, and LSP 235/17), and they were found inserted at multiple locations (Figure 1). It is of note that, of the total 38 IS*26* elements which have invaded the regions examined, only three of them were flanked by TSD, apparently derived from random insertion of IS*26* into particular genes, i.e., the *tnpA* gene of Tn*1721* in the SGI1-K variants of HUD 1/09 and HUD 2/09 (CGCTACCG), or the S025 *orf* belonging to the SGI1-P backbone of HUD 1/13 (GATAGCTA; although in this case the position and orientation of one of the TDS has been altered by further inversion). Insertion of IS*26* into *tnpA*_*Tn*_*_1721_* could have resulted from intermolecular copy in transposition, followed by resolution of the generated cointegrate through homologous recombination between the two directly oriented copies of the IS present in it. In contrast, the structure of HUD 1/13 could have originated by intramolecular copy in transposition of IS*26* into S025, using the *trans* pathway. Intramolecular copy in transposition events could also have been responsible for the generation of other inversions (*trans* attack) and deletions (*cis* attack) observed in the structures analyzed (He et al., 2015), while homologous recombination between oppositely oriented copies of IS*26* is likely to have originated the inversion of the *bla*_*TEM–*__1_ segment in the SGI1-K-like variant of LSP 150/10.

The *S*. Kentucky ST198-Cip^*R*^ clone is actively evolving, not only by altering the SGI1-K- and SGI-P-like structures, usually affecting their resistance gene content, but also through acquisition of plasmids, some of which encoding resistance to last resort antibiotics, like third generation cephalosporins and carbapenems (Hawkey et al., 2019). In the present study, plasmids with ColE, Col156, ColpVC, IncI1 or not identified replicons, were found in most isolates (69.2%). However, none of the detected plasmids were involved in resistance or conferred any other noticeable property to the carrier bacteria. In *S*. Heidelberg, which is a poultry-associated pathogen, like *S*. Kentucky ST198-Cip^*R*^ (Foley et al., 2011; Shah et al., 2017), carriage of ColE1 or ColpVC plasmids was shown to increase fitness of the bacteria in poultry litter (Oladeinde et al., 2018), and this might also be the case for the *S*. Kentucky isolates carrying such plasmids in the present study.

A global phylogenomic analysis of *S*. Kentucky ST198 has revealed that all MDR-Cip^*R*^ isolates carrying SGI1-K or variants herein belonged to a single monophylogenetic clade, and provided evidence that multiple independent transfers of this emergent pathogen out of Africa have occurred (Hawkey et al., 2019). The 13 *S*. Kentucky ST198-Cip^*R*^ isolates from Spanish hospitals, each carrying a distinct variant of SGI1-K, appear to belong to the African clone and were accordingly closely related. Interestingly, the closest isolates, HUD 1/13 and LSP 105/15, only differing by two SNP and carrying highly similar, but not identical SGI1-P variants, originated from hospitals placed in different regions of Spain, Basque Country and Asturias, respectively. Thus, after reaching Spain, the *S*. Kentucky ST198-CIP^*R*^ isolates are apparently spreading within the country, while their genomic islands continue to evolve, mainly as a consequence of the striking activity of IS*26*. As indicated before, such isolates belong to the high priority list of antibiotic-resistant bacteria compiled by WHO (Tacconelli et al., 2018), and so their continuous surveillance is required. The present study shows how implementation of WGS in clinical microbiology laboratories can efficiently help to conduct epidemiological studies of emerging pathogens, and to elucidate the genomic bases of antimicrobial drug resistance.

## Data Availability Statement

The datasets presented in this study can be found in online repositories. The names of the repository/repositories and accession number(s) can be found below: https://www.ncbi.nlm.nih.gov/genbank/, JACYBW000000000; https://www.ncbi.nlm.nih.gov/genbank/, JACYBX000000000; https://www.ncbi.nlm.nih.gov/genbank/, JACYBY000000000; https://www.ncbi.nlm.nih.gov/genbank/, JACYBZ000000000; https://www.ncbi.nlm.nih.gov/genbank/, JACYCA000000000; https://www.ncbi.nlm.nih.gov/genbank/, JACYBO000000000; https://www.ncbi.nlm.nih.gov/genbank/, JACYBP000000000; https://www.ncbi.nlm.nih.gov/genbank/, JACYBQ000000000; https://www.ncbi.nlm.nih.gov/genbank/, JACYBR000000000; https://www.ncbi.nlm.nih.gov/genbank/, JACYBS000000000; https://www.ncbi.nlm.nih.gov/genbank/, JACYBT000000000; https://www.ncbi.nlm.nih.gov/genbank/, JACYBU000000000; and https://www.ncbi.nlm.nih.gov/genbank/, JACYBV000000000.

## Ethics Statement

This study was reviewed and approved by the Research Ethics Committee of the Principality of Asturias (Code CEImPA 2020.446).

## Author Contributions

JF, MR, and RR designed the experiments. XV, MB, PL, MA, SH, EP, PI, and RR carried out the experiments. XV, MT, MR, and RR performed WGS analyses. XV, JF, MR, and RR drafted the manuscript. All authors approved the final version of this manuscript.

## Conflict of Interest

The authors declare that the research was conducted in the absence of any commercial or financial relationships that could be construed as a potential conflict of interest.

## Publisher’s Note

All claims expressed in this article are solely those of the authors and do not necessarily represent those of their affiliated organizations, or those of the publisher, the editors and the reviewers. Any product that may be evaluated in this article, or claim that may be made by its manufacturer, is not guaranteed or endorsed by the publisher.

## Footnotes

1
https://castillo.dicom.unican.es/upload/

2
https://www.ncbi.nlm.nih.gov/genome/annotation_prok/

3
https://cge.cbs.dtu.dk/services/
