# Supplementary material for: Genomic Analysis of Ciprofloxacin-Resistant Salmonella enterica Serovar Kentucky ST198 From Spanish Hospitals
Source: Front Microbiol. 2021 Oct 5;12:720449. doi: 10.3389/fmicb.2021.720449 (PMC8525328; doi:10.3389/fmicb.2021.720449)
Supplement: Supplementary file 1 [file Table_1.docx]

**Table S1**. Accession numbers of the genomes of *Salmonella enterica* serovar Kentucky ST198 isolates from Spanish hospitals, and parameters related to the quality of the assemblies

| **Isolate^a^** | **kmer** | **Contigs** | **N50** | **Longest contig**  **(bp)** | **Total bp**  **in contigs** | **Contigs**  **> 1 kb** | **Library** | **Coverage** | **GenBank accession number** |
| --- | --- | --- | --- | --- | --- | --- | --- | --- | --- |
| LSP 213/09 | 131 | 76 | 749,857 | 1,981,242 | 4,911,213 | 23 | 396 +/- 125 | 52x | JACYBW000000000 |
| LSP 150/10 | 131 | 98 | 773,753 | 2,003,827 | 4,843,533 | 21 | 321 +/- 106 | 38x | JACYBX000000000 |
| LSP 105/15 | 131 | 66 | 818,266 | 2,004,693 | 4,858,615 | 16 | 363 +/- 115 | 50x | JACYBY000000000 |
| LSP 235/17 | 131 | 65 | 818,144 | 2,004,440 | 4,859,250 | 17 | 383 +/- 122 | 87x | JACYBZ000000000 |
| LSP 314/17 | 131 | 66 | 773,796 | 2,000,029 | 4,798,087 | 14 | 341 +/- 120 | 53x | JACYCA000000000 |
| HUD 1/09 | 93 | 53 | 773,645 | 2,005,087 | 4,826,400 | 18 | 573 +/- 155 | 19x | JACYBO000000000 |
| HUD 2/09 | 89 | 65 | 773,539 | 2,003,673 | 4,832,977 | 18 | 586 +/- 155 | 30x | JACYBP000000000 |
| HUD 1/13 | 93 | 59 | 817,987 | 2,004,730 | 4,860,101 | 17 | 562 +/- 149 | 19x | JACYBQ000000000 |
| HUD 1/14 | 115 | 67 | 773,778 | 2,004,641 | 4,802,065 | 19 | 568 +/- 158 | 30x | JACYBR000000000 |
| HUD 1/15 | 93 | 62 | 773,572 | 2,004,640 | 4,827,143 | 19 | 574 +/- 155 | 19x | JACYBS000000000 |
| HUD 1/17 | 93 | 58 | 773,617 | 2,035,951 | 4,850,418 | 15 | 586 +/- 154 | 20x | JACYBT000000000 |
| HUA 3/18 | 89 | 69 | 773,590 | 1,210,202 | 4,825,331 | 22 | 495 +/- 122 | 20x | JACYBU000000000 |
| HUA 10/18 | 89 | 77 | 773,479 | 2,003,592 | 4,868,868 | 24 | 512 +/- 128 | 20x | JACYBV000000000 |

^a^, Isolates are designated with the initials of the center which supplied them, followed by a serial number/last two numbers of the year of recovery. LSP, “Laboratorio de Salud Pública”, Principality of Asturias, acting as regional reference center for *Salmonella*. LSP isolates come from “Hospital Universitario Central de Asturias”, Oviedo, Asturias (LSP 213/09 and LSP 105/15), “Hospital Universitario de Cabueñes”, Gijón, Asturias (LSP 150/10 and LSP 314/17) and “Hospital Universitario San Agustín”, Avilés, Asturias (LSP 235/17), HUD, “Hospital Universitario Donostia”, Basque Country; HUA, “Hospital Universitario de Álava”, Basque Country.

**Table S2.** Oligonucleotides used for the assembly of SGI1-K variants in *Salmonella enterica* serovar Kentucky isolates from Spanish hospitals

| **Primer name** | **Sequence (5’-3’)** | **Reference** |
| --- | --- | --- |
| *int*-SGI1-K | CTTGCGAAAAACACAGCCTC | this study |
| *S004*-R | ACAGAGATCATCTGCAGGTG | this study |
| *orfB-*F | CTGTGGGTTATCGTAGCTTC | this study |
| *orfB-*R | TCATTGGGAGTTAGACCACC | this study |
| *traG-*R | TATCCCTACCCAGATTTGCAGC | this study |
| *traH-S013* | TGAGTACAAACCGAACTCCC | this study |
| *S020*-R | TTGTCACATCGATGCGACCATG | this study |
| *S025*-F | TGCGTTAGTCTCGAGATGTG | this study |
| S*025*-R | ACTACCAGTCCAGTGATCTG | this study |
| *S026-*R | TTCTAGACGATGCTAGACCC | this study |
| *resG-R* | TTCTTCCCACTGGCCTTGTC | this study |
| i*ntI1_*In*4**-F | GTTACGACATTCGAACCGTGCAGG | this study |
| *intI1_*In*4*-*R | GGTCTGGTCATACATGTGATGG | this study |
| *qacEΔ1-*F | CAAGCTTTTGCCCATGAAGC | García et al., 2011 |
| IS*6100*-R | ATCGTTCCGTCCGTCCAATC | this study |
| *merC*-F | TTGGTTCAGTCATCGGCAATGG | this study |
| *tetA*-F | GCTACATCCTGCTTGCCT | García et al., 2011 |
| *tnpA_*Tn*1721*-F2 | ACTGGCTGCAAAGTGTTGAA | Martínez et al., 2007 |
| *tnpA_*Tn*1721*-R2 | TCCGTAAGGGCCGAAACTTC | Martínez et al., 2007 |
| *tnpA_*Tn*1721*-R4 | GAAGTTTCGGCCCTTACGGATG | this study |
| *strA*-R | CCAATCGCAGATAGAAGGC | García et al., 2011 |
| *tnpR_*Tn*3*-F | TCTTGAAGACGAAAGGGCCTCGTGAT | this study |
| *tnpR_*Tn*3*-R | GTATCACGAGGCCCTTTCGTCTTCAAGA | this study |
| *S044*-R | TCTTCGAGCGTCCAATGAAC | this study |
| *yidY*-R | CGGCACAATACAGGTAATCCCA | this study |

References

Garcia, P.; Guerra, B.; Bances, M.; Mendoza, M. C.; Rodicio, M. R. (2011). IncA/C plasmids mediate antimicrobial resistance linked to virulence genes in the Spanish clone of the emerging *Salmonella enterica* serotype 4,[5],12:i. *J Antimicrob Chemother* 66, (3), 543-549. doi: 10.1093/jac/dkq481

Martinez, N.; Mendoza, M. C.; Rodriguez, I.; Soto, S.; Bances, M.; Rodicio, M. R. (2007). Detailed structure of integrons and transposons carried by large conjugative plasmids responsible for multidrug resistance in diverse genomic types of *Salmonella enterica* serovar Brandenburg. *J Antimicrob Chemother* 60, (6), 1227-1234. doi: 10.1093/jac/dkm336

**Table S3.** Features and accession numbers of the genomes of *Salmonella enterica* serovar Kentucky ST198 isolates used for phylogenetic analysis in the present study

| **Isolate** | **Genome**  **size (bp)** | **Origin** | **Country** | **Year of**  **isolation** | **GenBank/ENA**  **accession no.** | **Reference/**  **Submitted by** |
| --- | --- | --- | --- | --- | --- | --- |
| pU131 | 4,900,326 | Human | USA | - | CP026327.1 | Unpublished |
| 161365 | 4,889,798 | Cattle isolate | Israel | - | CP043664.1 | Cohen et al., 2020 |
| 201001922 | 4,858,671 | Human (blood) | Morocco | - | CP028357.1 | Hawkey et al., 2019 |
| 201203105 | - | Human (stool) | Indonesia | - | SRR6898550 | Hawkey et al., 2019 |
| 162835 | 4,851,336 | Turkey isolate | Israel | - | CP043667.1 | Cohen et al., 2020 |
| K13SK002 | 4,847,949 | Chicken | South Korea | - | CP037917.1 | Unpublished |
| H5 | - | Chicken carcass | Egypt | - | PNYJ01000000 | USDA-ARS-USNPRC |
| H18 | - | Chicken carcass | Egypt | - | PNYI01000000 | USDA-ARS-USNPRC |
| NCTR281 | - | Rinse of chicken carcass | - | - | NQWT01000000 | FDA |
| 08-5707 | - | Human (stool) | Tanzania | 2008 | SRR6898500 | Hawkey et al., 2019 |
| 1090-10 | - | Turkey farm | Poland | 2010 | SRR6898559 | Hawkey et al., 2019 |
| 00-1059 | - | Human (urine) | Egypt | 2000 | SRR6898573 | Hawkey et al., 2019 |
| 01-2100 | - | Human (stool) | Egypt | 2001 | SRR6898513 | Hawkey et al., 2019 |
| 08-KS6 | - | Chicken | Nigeria | 2008 | SRR6898493 | Hawkey et al., 2019 |
| 201008553 | - | Human (stool) | Senegal | 2010 | SRR6898551 | Hawkey et al., 2019 |
| 07-1511 | - | Human (stool) | Morocco | 2007 | SRR6898518 | Hawkey et al., 2019 |
| 09-8391 | - | Human (stool) | Morocco | 2009 | SRR6898584 | Hawkey et al., 2019 |
| 80-11-309 | - | Human | Cambodia | 2011 | SRR6898581 | Hawkey et al., 2019 |

-, unknown

**References**

Cohen, E.; Davidovich, M.; Rokney, A.; Valinsky, L.; Rahav, G.; Gal-Mor, O. (2020). Emergence of new variants of antibiotic resistance genomic islands among multidrug-resistant *Salmonella enterica* in poultry. *Environ Microbiol* 22, (1), 413-432. doi: 10.1111/1462-2920.14858

Hawkey, J., Le Hello, S., Doublet, B., Granier, S.A., Hendriksen, R.S., Fricke, W.F.*, et al.* (2019). Global phylogenomics of multidrug-resistant *Salmonella enterica* serotype Kentucky ST198. *Microb Genom* 5. doi: 10.1099/mgen.0.000269

**Table S4.** Pairwise SNP distance matrix calculated from the genomes of 13 clinical isolates of *Salmonella enterica* serovar Kentucky from Spanish hospitals and 18 isolates from other countries and sources

|  | **00-1059** | **01-2100** | **07-1511** | **08-5707** | **08-KS6** | **09-8391** | **1090-10** | **161365** | **162835** | **201001922** | **201008553** | **201203105** | **80-11-309** | **H18** | **H5** | **HUA 1/09** | **HUA 1/16** | **HUD 1/09** | **HUD 1/13** | **HUD 1/14** | **HUD 1/15** | **HUD 1/17** | **HUD 2/09** | **K13SK002** | **LSP 105/15** | **LSP 150/10** | **LSP 213/09** | **LSP 235/17** | **LSP 314/17** | **NCTR281** | **pU131** |
| --- | --- | --- | --- | --- | --- | --- | --- | --- | --- | --- | --- | --- | --- | --- | --- | --- | --- | --- | --- | --- | --- | --- | --- | --- | --- | --- | --- | --- | --- | --- | --- |
| **00-1059** | 0 | 30 | 39 | 101 | 70 | 45 | 110 | 117 | 110 | 43 | 43 | 46 | 50 | 153 | 149 | 48 | 42 | 108 | 46 | 47 | 47 | 52 | 109 | 109 | 48 | 39 | 144 | 72 | 140 | 31 | 142 |
| **01-2100** | 30 | 0 | 35 | 97 | 66 | 41 | 106 | 113 | 106 | 39 | 39 | 42 | 46 | 149 | 145 | 44 | 38 | 104 | 42 | 43 | 43 | 48 | 105 | 105 | 44 | 35 | 140 | 68 | 136 | 27 | 138 |
| **07-1511** | 39 | 35 | 0 | 79 | 53 | 12 | 88 | 95 | 88 | 12 | 10 | 13 | 19 | 136 | 132 | 15 | 11 | 86 | 15 | 14 | 16 | 21 | 87 | 87 | 17 | 6 | 127 | 41 | 123 | 14 | 125 |
| **08-5707** | 101 | 97 | 79 | 0 | 116 | 85 | 15 | 22 | 15 | 83 | 81 | 86 | 90 | 73 | 69 | 88 | 82 | 13 | 86 | 87 | 87 | 92 | 14 | 14 | 88 | 79 | 64 | 112 | 64 | 77 | 62 |
| **08-KS6** | 70 | 66 | 53 | 116 | 0 | 59 | 125 | 132 | 125 | 57 | 57 | 60 | 64 | 167 | 163 | 62 | 56 | 123 | 60 | 61 | 61 | 66 | 124 | 124 | 62 | 53 | 158 | 86 | 150 | 45 | 156 |
| **09-8391** | 45 | 41 | 12 | 85 | 59 | 0 | 94 | 101 | 94 | 18 | 18 | 21 | 25 | 142 | 138 | 23 | 17 | 92 | 21 | 22 | 22 | 27 | 93 | 93 | 23 | 14 | 133 | 47 | 129 | 20 | 131 |
| **1090-10** | 110 | 106 | 88 | 15 | 125 | 94 | 0 | 25 | 18 | 92 | 92 | 95 | 99 | 82 | 78 | 97 | 91 | 22 | 95 | 96 | 96 | 101 | 23 | 21 | 97 | 88 | 73 | 121 | 73 | 86 | 71 |
| **161365** | 117 | 113 | 95 | 22 | 132 | 101 | 25 | 0 | 25 | 99 | 99 | 102 | 106 | 89 | 85 | 104 | 98 | 29 | 102 | 103 | 103 | 108 | 30 | 28 | 104 | 95 | 80 | 128 | 80 | 93 | 78 |
| **162835** | 110 | 106 | 88 | 15 | 125 | 94 | 18 | 25 | 0 | 92 | 92 | 95 | 99 | 82 | 78 | 97 | 91 | 22 | 95 | 96 | 96 | 101 | 23 | 21 | 97 | 88 | 73 | 121 | 73 | 86 | 71 |
| **201001922** | 43 | 39 | 12 | 83 | 57 | 18 | 92 | 99 | 92 | 0 | 16 | 19 | 23 | 140 | 136 | 21 | 15 | 90 | 19 | 20 | 20 | 25 | 91 | 91 | 21 | 12 | 131 | 45 | 127 | 18 | 129 |
| **201008553** | 43 | 39 | 10 | 81 | 57 | 18 | 92 | 99 | 92 | 16 | 0 | 11 | 23 | 140 | 136 | 13 | 15 | 90 | 19 | 18 | 20 | 25 | 91 | 91 | 21 | 10 | 131 | 45 | 127 | 18 | 129 |
| **201203105** | 46 | 42 | 13 | 86 | 60 | 21 | 95 | 102 | 95 | 19 | 11 | 0 | 26 | 143 | 139 | 6 | 18 | 93 | 22 | 21 | 23 | 28 | 94 | 94 | 24 | 13 | 134 | 48 | 130 | 21 | 132 |
| **80-11-309** | 50 | 46 | 19 | 90 | 64 | 25 | 99 | 106 | 99 | 23 | 23 | 26 | 0 | 147 | 143 | 28 | 22 | 97 | 26 | 27 | 27 | 32 | 98 | 98 | 28 | 19 | 138 | 52 | 134 | 25 | 136 |
| **H18** | 153 | 149 | 136 | 73 | 167 | 142 | 82 | 89 | 82 | 140 | 140 | 143 | 147 | 0 | 26 | 145 | 139 | 80 | 143 | 144 | 144 | 149 | 81 | 81 | 145 | 136 | 29 | 169 | 61 | 128 | 23 |
| **H5** | 149 | 145 | 132 | 69 | 163 | 138 | 78 | 85 | 78 | 136 | 136 | 139 | 143 | 26 | 0 | 141 | 135 | 76 | 139 | 140 | 140 | 145 | 77 | 77 | 141 | 132 | 25 | 165 | 57 | 124 | 19 |
| **HUA 1/09** | 48 | 44 | 15 | 88 | 62 | 23 | 97 | 104 | 97 | 21 | 13 | 6 | 28 | 145 | 141 | 0 | 20 | 95 | 24 | 23 | 25 | 30 | 96 | 96 | 26 | 15 | 136 | 50 | 132 | 23 | 134 |
| **HUA 1/16** | 42 | 38 | 11 | 82 | 56 | 17 | 91 | 98 | 91 | 15 | 15 | 18 | 22 | 139 | 135 | 20 | 0 | 89 | 18 | 19 | 17 | 22 | 90 | 90 | 20 | 11 | 130 | 44 | 126 | 17 | 128 |
| **HUD 1/09** | 108 | 104 | 86 | 13 | 123 | 92 | 22 | 29 | 22 | 90 | 90 | 93 | 97 | 80 | 76 | 95 | 89 | 0 | 93 | 94 | 94 | 99 | 19 | 21 | 95 | 86 | 71 | 119 | 71 | 84 | 69 |
| **HUD 1/13** | 46 | 42 | 15 | 86 | 60 | 21 | 95 | 102 | 95 | 19 | 19 | 22 | 26 | 143 | 139 | 24 | 18 | 93 | 0 | 23 | 23 | 28 | 94 | 94 | 2 | 15 | 134 | 30 | 130 | 21 | 132 |
| **HUD 1/14** | 47 | 43 | 14 | 87 | 61 | 22 | 96 | 103 | 96 | 20 | 18 | 21 | 27 | 144 | 140 | 23 | 19 | 94 | 23 | 0 | 24 | 29 | 95 | 95 | 25 | 14 | 135 | 49 | 131 | 22 | 133 |
| **HUD 1/15** | 47 | 43 | 16 | 87 | 61 | 22 | 96 | 103 | 96 | 20 | 20 | 23 | 27 | 144 | 140 | 25 | 17 | 94 | 23 | 24 | 0 | 27 | 95 | 95 | 25 | 16 | 135 | 49 | 131 | 22 | 133 |
| **HUD 1/17** | 52 | 48 | 21 | 92 | 66 | 27 | 101 | 108 | 101 | 25 | 25 | 28 | 32 | 149 | 145 | 30 | 22 | 99 | 28 | 29 | 27 | 0 | 98 | 100 | 30 | 21 | 140 | 54 | 136 | 27 | 138 |
| **HUD 2/09** | 109 | 105 | 87 | 14 | 124 | 93 | 23 | 30 | 23 | 91 | 91 | 94 | 98 | 81 | 77 | 96 | 90 | 19 | 94 | 95 | 95 | 98 | 0 | 22 | 96 | 87 | 72 | 120 | 72 | 85 | 70 |
| **K13SK002** | 109 | 105 | 87 | 14 | 124 | 93 | 21 | 28 | 21 | 91 | 91 | 94 | 98 | 81 | 77 | 96 | 90 | 21 | 94 | 95 | 95 | 100 | 22 | 0 | 96 | 87 | 72 | 120 | 72 | 85 | 70 |
| **LSP 105/15** | 48 | 44 | 17 | 88 | 62 | 23 | 97 | 104 | 97 | 21 | 21 | 24 | 28 | 145 | 141 | 26 | 20 | 95 | 2 | 25 | 25 | 30 | 96 | 96 | 0 | 17 | 136 | 32 | 132 | 23 | 134 |
| **LSP 150/10** | 39 | 35 | 6 | 79 | 53 | 14 | 88 | 95 | 88 | 12 | 10 | 13 | 19 | 136 | 132 | 15 | 11 | 86 | 15 | 14 | 16 | 21 | 87 | 87 | 17 | 0 | 127 | 41 | 123 | 14 | 125 |
| **LSP 213/09** | 144 | 140 | 127 | 64 | 158 | 133 | 73 | 80 | 73 | 131 | 131 | 134 | 138 | 29 | 25 | 136 | 130 | 71 | 134 | 135 | 135 | 140 | 72 | 72 | 136 | 127 | 0 | 160 | 52 | 119 | 18 |
| **LSP 235/17** | 72 | 68 | 41 | 112 | 86 | 47 | 121 | 128 | 121 | 45 | 45 | 48 | 52 | 169 | 165 | 50 | 44 | 119 | 30 | 49 | 49 | 54 | 120 | 120 | 32 | 41 | 160 | 0 | 156 | 47 | 158 |
| **LSP 314/17** | 140 | 136 | 123 | 64 | 150 | 129 | 73 | 80 | 73 | 127 | 127 | 130 | 134 | 61 | 57 | 132 | 126 | 71 | 130 | 131 | 131 | 136 | 72 | 72 | 132 | 123 | 52 | 156 | 0 | 115 | 50 |
| **NCTR281** | 31 | 27 | 14 | 77 | 45 | 20 | 86 | 93 | 86 | 18 | 18 | 21 | 25 | 128 | 124 | 23 | 17 | 84 | 21 | 22 | 22 | 27 | 85 | 85 | 23 | 14 | 119 | 47 | 115 | 0 | 117 |
| **pU131** | 142 | 138 | 125 | 62 | 156 | 131 | 71 | 78 | 71 | 129 | 129 | 132 | 136 | 23 | 19 | 134 | 128 | 69 | 132 | 133 | 133 | 138 | 70 | 70 | 134 | 125 | 18 | 158 | 50 | 117 | 0 |
